# Mechanistic Insights Into the Immune Pathophysiology of COVID-19; An In-Depth Review

**DOI:** 10.3389/fimmu.2022.835104

**Published:** 2022-03-24

**Authors:** Areez Shafqat, Shameel Shafqat, Sulaiman Al Salameh, Junaid Kashir, Khaled Alkattan, Ahmed Yaqinuddin

**Affiliations:** ^1^ College of Medicine, Alfaisal University, Riyadh, Saudi Arabia; ^2^ Medical College, Aga Khan University, Karachi, Pakistan; ^3^ Center of Comparative Medicine, King Faisal Specialist Hospital and Research Centre, Riyadh, Saudi Arabia

**Keywords:** Coronavirus, immunopathogenesis, pathophysiology, protective immunity, vaccine

## Abstract

Severe Acute Respiratory Syndrome Coronavirus-2 (SARS-CoV-2), which causes coronavirus-19 (COVID-19), has caused significant morbidity and mortality globally. In addition to the respiratory manifestations seen in severe cases, multi-organ pathologies also occur, making management a much-debated issue. In addition, the emergence of new variants can potentially render vaccines with a relatively limited utility. Many investigators have attempted to elucidate the precise pathophysiological mechanisms causing COVID-19 respiratory and systemic disease. Spillover of lung-derived cytokines causing a cytokine storm is considered the cause of systemic disease. However, recent studies have provided contradictory evidence, whereby the extent of cytokine storm is insufficient to cause severe illness. These issues are highly relevant, as management approaches considering COVID-19 a classic form of acute respiratory distress syndrome with a cytokine storm could translate to unfounded clinical decisions, detrimental to patient trajectory. Additionally, the precise immune cell signatures that characterize disease of varying severity remain contentious. We provide an up-to-date review on the immune dysregulation caused by COVID-19 and highlight pertinent discussions in the scientific community. The response from the scientific community has been unprecedented regarding the development of highly effective vaccines and cutting-edge research on novel therapies. We hope that this review furthers the conversations held by scientists and informs the aims of future research projects, which will potentially further our understanding of COVID-19 and its immune pathogenesis.

## 1 Introduction

The global pandemic of Coronavirus-19 (COVID-19), caused by Severe Acute Respiratory Syndrome Coronavirus 2 (SARS-CoV-2), has currently stands at over 382,177,997 million reported cases, with 5.7 million deaths. Over the past year, an unprecedented effort from the scientific community has yielded highly effective vaccines presently in use; the FDA approves the Pfizer Bio-NTech and Moderna mRNA vaccines and the Janssen (Johnson and Johnson) viral vector vaccine (1).

However, newly emerging variants, termed variants of concern (VOCs), represent a significant threat to the efficacy of currently employed vaccines, exemplified by the most recently identified Omicron variant (2). The diverse clinical manifestations of COVID-19 also hinder effective management. In addition to the well-documented respiratory presentations, multi-organ pathologies occur in critical COVID-19 patients. Furthermore, emerging evidence highlights the long-lasting complications of COVID-19 after acute disease, called post-acute COVID-19 sequelae (PACS) or long COVID. The pathophysiology of such clinical complexities involves a dysregulated immune response, leading to a systemic cytokine storm, albeit with contraindications from various mounting pieces of evidence.

Gaining mechanistic insights into pathways elicited by SARS-CoV-2 infection could provide opportunities to optimize management protocols for improving the clinical outcomes of patients. Multiple reports recognize that numerous strategies tackle COVID-19 based on broad similarities between COVID-19-induced acute respiratory distress syndrome (ARDS) and other ARDS-causing diseases. This confusion could translate into unfounded decisions in the hospital, detrimental to patient outcomes. Although in principle, COVID-induced ARDS shares commonalities with ARDS, emerging evidence strongly advocates that SARS-CoV-2 is entirely a unique pathologic entity (3).

This review provides an update regarding the infection and immune dysregulation mechanisms in COVID-19. However, our review will mainly discuss the relevant topics’ salient features due to the sheer mass of publications.

## 2 Anatomy of SARS-CoV-2 Infection and Clinical Features

### 2.1 Mechanisms of SARS-CoV-2 Infection

SARS-CoV-2 enters host cells by the surface S protein comprising S1 and S2. The S1 subunit contains the receptor-binding domain (RBD), whereas S2 mediates viral-cell membrane fusion and cell entry (1). After engaging its cognate receptor, which is the angiotensin-converting enzyme 2 (ACE2) receptor, the S-protein undergoes priming through proteolytic cleavage by transmembrane serine protease-2 (TMPRSS2), which induces a conformational change in S-protein and allows for cellular entry *via* endocytosis (1). The distribution of ACE2 throughout the body dictates SARS-CoV-2 infective tropism. In the respiratory zone of the airways, type II pneumocytes mainly express ACE2, hence constituting the primary target of SARS-CoV-2 in the alveoli (4). A study revealed a decreasing gradient of ACE2 expression from the upper to the lower respiratory tract with a corresponding decline in SARS-CoV-2 viral load. The question in response is how SARS-CoV-2 gets transmitted from the upper to the lower airways? The same study highlighted aspiration-mediated viral seeding of the lower respiratory tract as a potential mechanism, which leads to infection of type II pneumocytes, alveolar macrophages, and endothelial cells expressing ACE2 (5).

Cardiac, kidney, gastrointestinal, bile duct, and testicular cells also express ACE2, rendering them susceptible to infection and cytopathic effects (6). Moreover, SARS-CoV-2 RNA and proteins have been detected in the brain, raising suspicion of neurotropism (7) manifesting clinically as anosmia or/and ageusia, which indeed are central features of COVID-19 infection. In addition, demonstration of SARS-CoV-2 elements in the small bowel after clinical recovery suggests that SARS-CoV-2 persists in the gastrointestinal tract, even after recovery. Stool shedding of SARS-CoV-2 occurs, raising concern for potential fecal-oral transmission (6). Although not definitively proven yet, such possible routes of SARS-CoV-2 acquisition should be considered and eliminated in hospital environments to prevent nosocomial infection.

Although SARS-CoV-2 infection is less severe than the original SARS-CoV and Middle East Respiratory Syndrome (MERS)-CoV, the increased transmissibility of SARS-CoV-2 is responsible for the increased morbidity and mortality worldwide compared to other beta-coronaviruses. This increased transmissibility stems from SARS-CoV-2 replication in upper respiratory epithelial cells and subsequent nasal and pharyngeal shedding, features not exhibited by SARS-CoV and MERS-CoV. SARS-CoV-2 viral load peaks at about 3-5 days after infection, whereas loads of SARS-CoV and MERS-CoV are maximal after approximately ten days post-symptom onset (8–10). MERS-CoV can also directly infect innate immune cells to augment viral replication, whereas SARS-CoV exhibits abortive infection of these cells. Conclusions regarding potential immune cell infection by SARS-CoV-2 would be premature due to the paucity of current evidence. Further work evaluating SARS-CoV-2 immune cell infection is required to provide a definitive answer. Only two such demonstrations – one in preprint form – exist in current literature, reporting monocyte infection in the alveolar spaces and secondary lymphoid organs (10, 11).

ACE2 is unlikely the only receptor mediating SARS-CoV-2 cell entry. A study identified 12 additional receptor types facilitating SARS-CoV-2 infection independent of ACE2. Perhaps the expression of such receptors accounts for the broad SARS-CoV-2 tropism and the variable clinical manifestations of COVID-19 (12). For example, Neuropilin-1 binds to the C-end rule (CendR) peptide at the C-terminal end of S1 after proteolytic cleavage of the SARS-CoV-2 S protein. Although the binding affinity between CendR and neuropilin-1 is considerably weaker than RBD-ACE2 interactions, the nasopharynx and upper respiratory tract express neuropilin-1 more abundantly than ACE2 (13). Another receptor, the tyrosine kinase UFO termed AXL, could mediate ACE2-independent SARS-CoV-2 viral entry into pulmonary epithelial cells by binding the S-protein N-terminal domain (NTD) rather than the RBD (14). Like neuropilin-1, AXL is more abundantly expressed in the respiratory tract than ACE2, with the binding affinity of AXL-NTD interactions comparable to ACE2 and expression levels of AXL correlating with SARS-CoV-2 viral load in bronchoalveolar lavage fluid (BALF) (14). Accordingly, blocking AXL-NTD interactions in ACE2-depleted H1299 cells abolishes viral entry, and blocking ACE2 or AXL while overexpressing the other receptor has minimal effects.

These results collectively implicate AXL as the primary receptor alongside ACE2 mediating SARS-CoV-2 infection (14). Therefore, future research should evaluate whether similar infectious processes result from the engagement of these receptors. Furthermore, assessing the expression of AXL and other receptors on remote tissues and assessing for viral SARS-CoV-2 load despite ACE2 inhibition would implicate these alternate receptors as playing central roles in the diverse clinical presentation of COVID-19. If this is the case, then AXL could constitute a novel therapeutic target for future antiviral COVID-19 medications to mitigate the systemic manifestations of COVID-19. Finally, several other receptors are potentially responsible for the high infectivity of SARS-CoV-2 and thus require similar investigations to substantiate their roles in COVID-19 pathogenesis (14).

### 2.2 Clinical Features

The pulmonary tropism of SARS-CoV-2 manifests in the most common clinical presentation of COVID-19, cough and dyspnea (15). Although ~80% of COVID-19 patients are asymptomatic or develop mild flu-like symptoms, approximately 15% progress to critical illness characterized by ARDS requiring mechanical ventilation and aggressive treatment in intensive care units (ICU) (3, 16). From a functional standpoint, SARS-CoV-2 likely generates a ventilation-perfusion mismatch (V/Q mismatch) through shunting and increasing dead space ventilation by causing inflammation-induced pulmonary hyperperfusion and microvascular thrombosis, respectively. The consequent pulmonary edema thickens the alveolar-pulmonary capillary diffusion barrier, impeding efficient gas exchange (3, 17).

Early COVID-19 manifests predominantly as bilateral, subpleural, or peripheral ground-glass opacities (GGOs) on computed tomography (CT) scans. GGOs denote airway disease characterized by the partial filling of the alveolar air spaces with concomitant interstitial thickening, inflammation, and edema, all typical features of a pneumonia pattern (18, 19). Such findings peak at 9-13 days and subsequently begin to resolve. However, CT scans can reveal increasing disease severity by increasing consolidation, a crazy-paving appearance (GGOs with superimposed thickening of the inter-and intralobular septae), and a more global lung involvement (18).

Rapid respiratory deterioration in patients with relatively mild lung disease, with abnormal coagulation parameters, and right heart failure are suggestive of pulmonary embolism (PE), for which a pulmonary CT angiogram (CTPA) is indicated (20). Clinical studies have shown a high prevalence of PE in critically ill COVID-19: the composite incidence of venous thromboembolism and arterial thrombotic complications in ICU-admitted COVID-19 pneumonia patients is 31% (21), and 20.6% of ICU-admitted COVID-19 patients develop PE with an absolute higher risk (14.4%) compared to non-COVID-related ICU admissions (22). In agreement with this, autopsy findings of COVID-19 lung disease reveal vascular microangiopathy and thrombosis, setting this disease apart from ARDS secondary to other causes (23).

After the resolution of severe COVID-19, lung fibrosis can develop, resulting in a progressive and irreversible deterioration in lung function and respiratory failure. Fibrosis is a confirmed complication of severe SARS infection, with the severity dependent on disease duration; prior studies reporting pulmonary autopsy findings of severe COVID-19 have revealed extensive fibrotic changes (24). CT findings of such entities include extensive fibrotic changes with reticulations, traction bronchiectasis, and honeycombing (25). Predictors of post-COVID fibrosis – aptly named long COVID or PACS– include old age, male gender, comorbidities such as hypertension, diabetes mellitus, preexisting chronic pulmonary disease, and a longer duration of symptoms. Concurrently, these conditions are also risk factors for severe COVID-19 ARDS. Indeed, lung fibrosis is a well-known sequela of ARDS irrespective of etiology (25, 26).

Another distinguishing feature separating COVID-19 ARDS from other etiologies is the apparent silent hypoxemia – defined as minimal dyspnea out of proportion to the hypoxemia and lung damage – early in the disease course. Three distinct pathophysiological processes may underlie this: high lung compliance early in the disease course, impairment of hypoxic vasoconstriction resulting in shunting, and impairment of peripheral chemoreceptor oxygen-sensing sensitivity by direct SARS-CoV-2 infection (17). However, hypoxemia has minimal contribution to the severe dyspnea that COVID patients suffer. Instead, hypoxia-driven hyperventilation and an increased tidal volume seen in COVID-induced pneumonia can result in patient-inflicted lung injury. These patients exhibit patterns of lung injury approximating those seen in mechanical ventilation-associated injuries (27). Therefore, such patients may benefit from prophylactic rather than therapeutic protective ventilation (3, 27).

### 2.3 Comorbidities Linked to COVID-19 Severity

Numerous clinical studies have shown that individuals with pre-existing comorbidities are at the highest risk of severe disease (28). Two meta-analyses have shown that individuals with chronic comorbidities, including hypertension (16%), cardiovascular disease (12.11%), diabetes (7.87%), chronic liver (3%) and kidney disease (0.83%), cancer (0.92%), chronic obstructive pulmonary disease (0.95%), and cancer (0.92%), account for the majority of COVID-19 hospitalizations (29, 30). However, the factors underlying this predisposition remain investigational.

**Figure 1:**
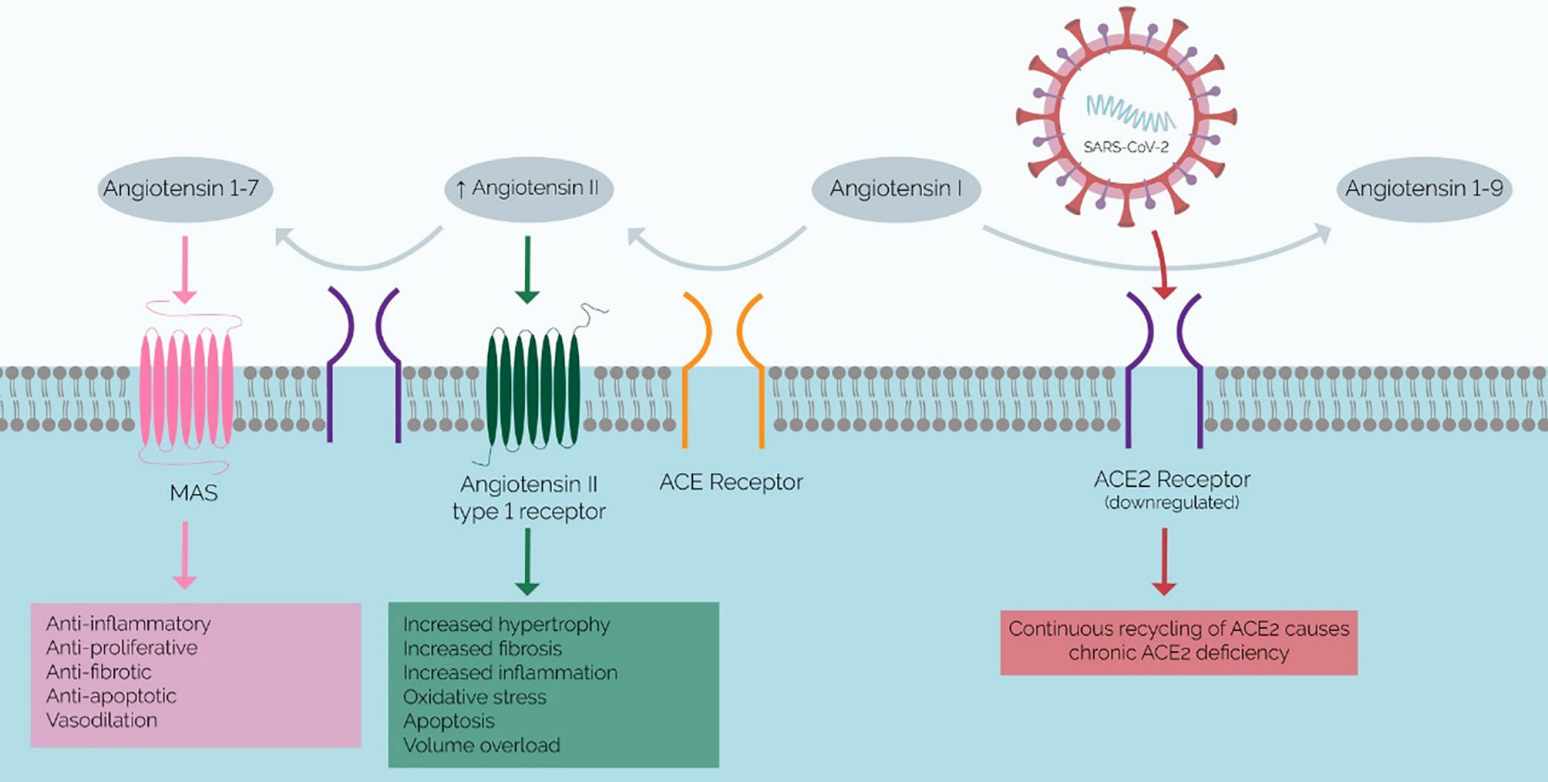
Mechanism of SARS-CoV-2 Infection and Proposed RAAS Imbalance. ACE2 (purple) converts angiotensin II to angiotensin 1-7, which has anti-inflammatory effects. The chronic ACE2 deficiency that results in COVID-19 results in a buildup of angiotensin II and a lack of angiotensin 1-7, which promotes inflammation and fibrosis. Therefore, this RAAS imbalance has been implicated in the pathophysiology of the systemic inflammatory phenotype of severe COVID-19.

These comorbidities induce renin-angiotensin-aldosterone (RAS) imbalance, and SARS-CoV-2 exacerbates this. Elevated Ang II is a prominent feature of various comorbidities – such as diabetes, obesity, and hypertension – and may account for the elevated risk of severe COVID-19 in these patients (31, 32) SARS-CoV-2 utilizes ACE2 for viral entry into cells and the continuous recycling of ACE2 upon SARS-CoV-2 cell entry – i.e., repetitive endocytosis and recirculation back to the cell surface – eventually results in a net downregulation of ACE2 and a chronic ACE2 deficiency (33). ACE2 converts Angiotensin II (Ang-II) to Angiotensin 1-7 (Ang1-7). Therefore, ACE2 deficiency causes Ang II accumulation. Ang II and Ang1-7 thus have reciprocal functions: ACE2 promotes proinflammatory and prothrombotic phenotypes through the angiotensin-1 receptor (AT1R), while Ang1-7 mediates the opposite (34, 35). Thus, increases in serum Ang II contribute to the proinflammatory and thrombotic phenotype of COVID-19 (35) (**Figure 1**). Lastly, since the ACE2 gene is X-linked, some studies hypothesize this to underpin the apparent elevated disease severity in males, i.e., reduced ACE2 expression occurs in males [15,32]. Additionally, reduced ACE2 expression with age may contribute to the high risk of severe disease in the elderly (4).

If ACE2 expression levels do inversely correlate with disease severity, coupled with the current need for reliable prognostic biomarkers, perhaps scrutinizing ACE2 levels as potential biomarkers could answer these pertinent clinical questions.

## 3 Innate Immune Responses Against SARS-CoV-2

**Figure 2:**
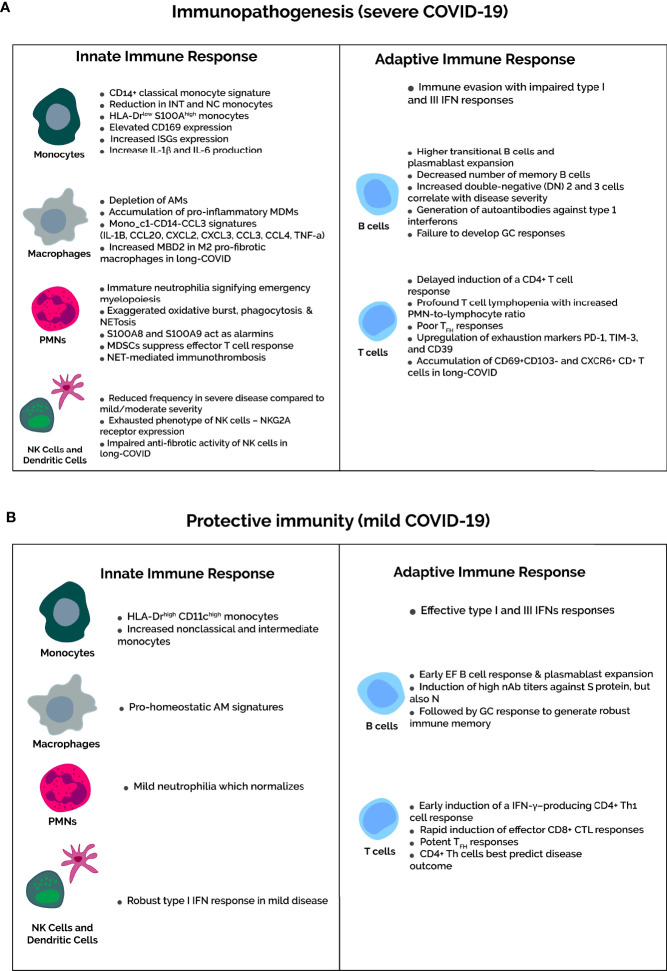
Severity-dependent immune profiles upon SARS-CoV-2 infection. **(A)** In severe COVID-19, the immune evasion capabilities of SARS-CoV-2 inhibits IFN responses to delay the recruitment of functional T cells. Consequently, dysfunctional T cell immunity occurs in severe cases, marked by severe lymphopenia and higher expression of T cell exhaustion markers PD-1, TIM-3, and CD39. Not mentioned in the diagram is the increased number of Tregs in severe cases. Concomitantly, amplification of the innate response is characterized by accumulation of classical HLA-DR^low^S100A^high^ proinflammatory monocytes and depletion of pro-homeostatic alveolar macrophages. Regarding neutrophils, an immature neutrophilia suggests emergency myelopoiesis, and neutrophil activation markers, such as oxidative burst, phagocytosis, and NETosis, increase in severe COVID-19. In addition, the MDSCs that contribute to the neutrophilia suppress T-cell responses and activate Tregs. Composition rather than quantity of the B cell compartment are altered in severe COVID-19, featuring more antibody-secreting plasmablasts and impaired germinal center responses with a decrease in memory B cells and T_FH_ cells. **(B)** In contrast, mild COVID-19 infection is associated with recovery of T cell counts and function. The neutrophilia and HLA-DR expression on monocytes normalizes in mild cases. This is due to early induction of IFN responses upon SARS-CoV-2 infection, which elicits an effective and timely T-cell response. B cell compartment modifications are as expected for viral infections, with potent early plasmablast responses and subsequent GC responses to yield long-lived SARS-CoV-2-specific plasma and memory cells.

Innate immunity is the first line of defense against invading microbes and involves a nonspecific immune response with neutrophil and macrophage recruitment and cytokine and chemokine production. Immunologically profiling Mild-to-moderate versus severe COVID-19 reveals strikingly different, severity-dependent innate and adaptive immune responses (**Figure 2**). The innate response also kickstarts adaptive immunity through antigen-presenting cells (APCs) – namely macrophages and dendritic cells – which present viral epitopes to CD4+ T helper cells.

The innate immune system responds to SARS-CoV-2 infection *via* two mechanisms: (1) directly by recognizing pathogen-associated molecular patterns (PAMPs) *via* pattern recognition receptors (PRRs) – including NOD-like (NLR), RIG-like (RLR), and Toll-like (TLR) receptors – present on and in immune cells and (2) indirectly through the release of cell contents – termed damage-associated molecular patterns (DAMPs) – which PRRs recognize (4).

Briefly, host defense comprises various pattern recognition molecules residing in all body compartments. Membrane-bound PRRs include Toll-like receptors (TLRs) and C-type lectin receptors (CLRs). Cytoplasmic PRRs include melanoma differentiation-associated gene 5 (MDA5), retinoic acid-inducible gene I (RIG-I), Z-DNA binding protein 1 (ZBP1), and cyclic GMP-AMP synthetase (cGAS). In the extracellular compartment, complement through mannan-binding lectin (MBL) can sense viral glycans to induce the lectin pathway of complement activation. Non-classical PRRs include the stress-induced nuclear factor erythroid 2-related factor 2 (Nrf2) and hypoxia-inducible factor 1α (HIF1α), activated in response to oxidative stress and hypoxia, respectively (36). These PRRs induce transcriptional programs to activate either inflammatory or antiviral gene expression. For instance, NFκB and activator protein 1 (AP1) drive proinflammatory gene expression signatures, whereas IFN regulatory factor 3 (IRF3) and (IRF7) drive antiviral type I and III IFN signatures (37, 38).

Specifically, type I IFN expression is critical in clearing SARS-CoV-2. Indeed, like other coronaviruses, animal models of SARS-CoV-2 infection show the virus to be highly sensitive to type I IFN treatment. Therefore, this pathway represents an essential immune-evasion mechanism of SARS-CoV-2. For instance, several above-described PRR-mediated type I IFN antiviral programs described above are suppressed explicitly in SARS-CoV-2 infection. Furthermore, the data indicate that molecular defects and consequent dysregulations in generating interferon (IFN) responses elevate the risk of severe COVID-19 (39, 40). Lastly, SARS-CoV-2 delays type I IFN expression in *in-vitro* studies. For more detailed descriptions on this topic, we refer readers to a recently published comprehensive review solely focusing on innate immunological pathways in COVID-19 (41).

This section focuses on neutrophil and monocyte/macrophage responses. However, COVID-19 also disrupts other components of innate immunity, namely natural killer (NK) cells and dendritic cells (DCs). Briefly, total DCs frequency decreases in severe COVID-19 patients compared to those mild-to-moderately affected and healthy controls (42). Whereas mild COVID-19 involves robust type I IFN responses early in the disease course, severe disease impairs type I IFN response and delays the development of adaptive immunity with consequent amplification of the innate response (43). Delayed activation of adaptive T cell responses is crucial to COVID-19 disease progression.

Studies report a reduction in NK cells in the periphery but their accumulation in the lungs. NK cell activation signatures in COVID-19 are controversial. Some studies report an exhausted phenotype with higher NKG2A expression and reduced makers cytotoxicity (44, 45). At the same time, other investigators show marked peripheral NK cell activation in severe COVID-19, with higher fractions of CD56^bright^ cells and higher cytotoxic makers perforin and granzyme B that correlate positively with serum IL-6 levels, neutrophilia, sequential organ failure assessment (SOFA) scores, and PaO_2_/FiO_2_ ratio (46). Nevertheless, NK cell dysfunction is a feature of COVID-19 and correlates with disease severity, and a conditional independence network analysis revealed this to be IL-15-dependent (47). Lastly, NK cells in severe COVID-19 patients have impaired antifibrotic activity (48), potentially setting the stage for PACS.

### 3.1 Neutrophil, Monocyte, and Macrophage Responses in COVID-19

#### 3.1.1 Neutrophils

Neutrophils are the first cells to be recruited to sites of acute inflammation and contribute to inflammatory responses by phagocytosis, killing microbes *via* respiratory bursts, degranulating to release antimicrobial peptides, and elaborating neutrophil extracellular traps (NETs). All severities of COVID-19 consistently feature neutrophilia and elevated neutrophil activation markers, including oxidative burst, NETosis, and phagocytosis relative to healthy controls, and these findings correlate with disease severity (49, 50). Single-cell RNA sequencing analyses reveal dense neutrophilic infiltrates in the upper airways (51) and BALF (52), and histopathological investigations confirm these findings by showing excessive neutrophil infiltration into the lungs of deceased COVID-19 patients (53). BALF analysis also reveals higher levels of neutrophil-associated cytokines and chemokines IL-6 and IL-8, respectively. The neutrophilia in critically ill COVID-19 patients is skewed towards immature neutrophil subsets, suggesting emergency myelopoiesis. Transcriptomic analysis of immature neutrophils in severe COVID-19 reveals higher expression of genes involved in neutrophil extracellular trap (NET) formation, such as MPO, ELANE, PRTN3, and genes associated with a poor outcome in sepsis (54). Additionally, neutrophils in COVID-19 express high S100A8 and S100A9, which serve as alarmins to amplify inflammation (55).

The neutrophilia in severe disease also shows myeloid-derived suppressor cells (MDSCs), more so in ICU patients compared to milder cases, and in non-survivors compared to survivors. Furthermore, higher MDSCs correlate with increased IL-6 levels in non-survivors and lower T cell proliferation and IFN- production (56). Therefore, MDSCs play a role in dampening T cell immunity (57). Accordingly, the current data suggest a model whereby an early expansion of MDSCs can impair protective T cell responses and cause disease progression (58), rationalizing MDSC profiling as a potential risk factor in prognosis. Furthermore, another study reported MDSCs as the primary source of IL-6 in severe COVID-19, but administering tocilizumab (an IL-6 inhibitor) did not reduce circulating MDSC or IL-6 levels. Lastly, MDSCs can induce expansion of regulatory T cells (Tregs), which are characteristically elevated in COVID-19, suggesting a causal relationship between MDSCs and Tregs in COVID-19 (58, 59).

#### 3.1.2 Monocyte Responses in COVID-19

The mononuclear phagocyte (MNP) system is composed of monocytes, macrophages, and dendritic cells (DCs). Monocytes derive from hematopoietic myeloid progenitors in the bone marrow and enter the circulation. In tissues, monocytes differentiate into DCs or macrophages. The relative surface expression of CD14 and CD16 on monocytes divide them into three subsets: classical (CD14^++^CD16^-^), intermediate (CD14^+^CD16^+^), and non-classical (CD14^+^CD16^++^) (60). Classical monocytes are the most abundant, while nonclassical monocytes are the least common. In general, classical monocytes are proinflammatory, intermediate monocytes possess robust antigen-presenting and cytokine-producing capabilities, and nonclassical monocytes maintain vascular integrity and initiate antiviral responses by elaborating type I IFN responses (61, 62). Therefore, the subsets of circulating monocytes are phenotypical and functionally distinct (63), and each subset is differentially affected by SARS-CoV-2 infection (64, 65).

Severe SARS-CoV-2 infection-induced disturbances to the monocyte compartment take three forms: monocytopenia with depletion of nonclassical monocytes, reduced HLA-DR and CD11c expression, and increased expression of the S100A family genes (43, 55, 66–69). Therefore, whereas mild COVID-19 features HLA-DR^high^ CD11c^high^ monocytes, severe disease features HLA-DR^low^ S100A^high^ classical monocytes showing signs of activation such as elevated CD169 and CD163 expression, upregulation of ISGs, and increased release of alarmins and proinflammatory cytokines IL-1β and IL-6 (70, 71). In agreement with this, moderate COVID cases show an increased percentage of intermediate monocytes. Furthermore, longitudinally profiling monocyte subset alterations in convalescent COVID-19 reveals a decline in the percentage of classical monocytes and their activation markers with a concurrent rise in intermediate and nonclassical monocytes fractions (72). 

Single-cell RNA sequencing (scRNA-seq) shows classical monocytes as the primary source of cytokines and chemokines in severe COVID-19, such as CCL2, CXCL8, IL-6, TNF-α. IL-1β, and IL-18 (73). Another study profiled the blood to reveal three distinct monocyte subtypes: Mono_c1-CD14-CCL3, which accumulated in a subset of severely affected patients with cytokine storm, Mono_c2-CD14-HLA-DPB, and Mono_c3-CD14-VCAN, both of which were present to comparable levels in every disease stage (74). Also, Mono_c1-CD14-CCL3 monocyte-derived CCL3, IL-1RN, and TNF correlate with disease severity (74). Lastly, as discussed below, monocytes are recruited into the lungs in COVID-19 patients (75).

#### 3.1.3 Macrophage Responses in COVID-19

Although immunologically profiling whole blood and PBMCs has been invaluable in ascertaining the systemic immune dysregulation caused by SARS-CoV-2 infection, COVID-19 is, in the end, a respiratory disease. Therefore, analyzing local immune responses in the lung is essential to understanding the immunopathology of COVID-19. Lung-resident macrophages are classified based on exact location into alveolar macrophages (AMs), monocyte-derived macrophages (MDMs), and transitioning MDMs. AMs reside in the alveolar lumen and orchestrate homeostasis and anti-inflammatory responses, whereas MDMs propagate inflammation and fibrosis (76). AMs originate in the fetal yolk-sac, from where they migrate to the lungs and maintain themselves throughout life by self-renewal, whereas MDM populations are replenished by circulating monocytes.

The seminal study of Liao et al. compared cellular composition and immune profiles of BALF of COVID-19 patients of varying severities (52). Severe COVID-19 alters myeloid and lymphoid compartments: neutrophils, macrophages, and monocytes increase, whereas dendritic and T cells are depleted (52). Another study obtained paired blood and airway samples of severe COVID-19 patients, revealing T-cell depletion in the blood and lungs along with increased myeloid frequencies and a dense MDM and neutrophilic infiltrate. In addition, increased T cell numbers were seen in younger patients and correlated with better COVID-19 outcomes, whereas myeloid cell numbers were positively associated with age (77).

The BALF of critical COVID-19 patients features depletion of AMs and higher proportions of MDMs (52, 78). Whereas AMs exert pro-homeostatic effects, MDMs are proinflammatory, and, therefore, their accumulation contributes to the immunopathology of COVID-19 lung disease. A study in pre-print evaluating hospitalized COVID-19 patients also revealed a depletion of AMs, which correlated with disease severity, and AMs numerically and functionally normalized in recovering patients (77). Analysis of cytokine and chemokine expression levels revealed higher IL-1β, IL-6, TNF, and CCL2, CCL3, CCL4, and CCL7 in severe COVID-19 BALF compared to moderate disease. Furthermore, CXCL16, a chemokine attracting T cells, was depleted in severe COVID-19 but was induced in mild disease. In contrast, elevated CCL2 expression indicates a CCL2-CCR2 axis that recruits more myeloid cells into COVID-19 lungs (77). The IL-6 elevation has two-fold effects: it exacerbates the inflammatory response and reduces HLA-DR expression on monocytes. Accordingly, COVID-19 patient plasma inhibits HLA-DR expression on monocytes (56, 79, 80), and tocilizumab restores HLA-DR expression levels and non-classical monocytes (81).

Macrophages also contribute to pulmonary fibrosis, a cardinal feature of various diseases like idiopathic pulmonary fibrosis (IPF) and PACS. Macrophages are either polarized to a proinflammatory M1 phenotype or an anti-inflammatory pro-fibrotic M2 phenotype, depending on their specific tissue microenvironment. M2 macrophages contribute to pulmonary fibrosis by releasing TGFβ1 and PDGF, which, in turn, cause fibroblasts to differentiate into myofibroblasts which mediate fibrosis. A recent study demonstrated profibrotic MDM phenotypes in severe COVID-19, which were transcriptionally similar to macrophages found in IPF (82). Such profibrotic macrophages interact with fibroblasts and myofibroblasts to promote pulmonary fibrosis manifested as the aforementioned radiologic findings.

Additionally, SARS-CoV-2-infected monocytes exhibited a profibrotic transcriptome and proteome approximating monocyte signatures in IPF, which uninfected cells do not possess (82). A recent study demonstrated increased methyl-CpG-binding domain protein (MBD) 2 in lung M2 macrophages in COVID-19, IPF, systemic sclerosis-induced interstitial lung disease, and in mice following bleomycin-induced pulmonary fibrosis (83). MDB2 enhances PI3K/Akt signaling to induce TGFβ1 production and cause fibrosis. Notably, depletion of MDB2 in mice attenuates bleomycin-induced pulmonary fibrosis by reducing TGF-β1 levels, and administering liposomal forms of MBD2 siRNA protects mice from bleomycin-induced pulmonary fibrosis (83). Therefore, it will be intriguing to see how future research exploits the potential role of MDB2 in COVID-19 pulmonary fibrosis to alleviate symptoms of PACS. Intriguingly, another recent study demonstrated the persistence of SARS-CoV-2 S1 protein in nonclassical monocytes up to 15 months post-infection, which could account for the heterogenous presentation of long COVID, whereby nonclassical monocytes exploit their function of maintaining vascular integrity to migrate to various tissues and cause pathology (64).

#### 3.1.4 Metabolic Reprogramming in COVID-19

Since viruses are obligate intracellular pathogens, they rely solely on host cell machinery for survival, i.e., translation of viral proteins and production of progeny virions. Therefore, viruses tend to disrupt host cell metabolism to maximize their survival. This metabolic reprogramming contributes to determining the clinical outcome of a viral infection, conferring to it prognostic and therapeutic importance. SARS-CoV-2 also brings about extensive metabolic reprogramming (84).

Most differentiated cells utilize aerobic metabolism, i.e., oxidative phosphorylation, for energy in the form of ATP. Viruses use HIF1α, which upregulates enzymes of glycolysis, to redirect cell metabolism into anaerobic glycolysis to generate the ATP necessary for viral replication (85). SARS-CoV-2 also upregulates glycolysis in host immune cells *via* HIF1α, as evidenced by elevated pyruvate, pyruvate kinase, and lactate dehydrogenase (LDH) in COVID-19 patients (86). In addition, emergency myelopoiesis occurs in critically ill COVID-19 patients, with augmented expression of HIF1α and its associated transcriptional targets (87).

SARS-CoV-2 utilizes ORF3a to cause mitochondrial dysfunction and ROS production, which activate HIF1α (88). Furthermore, HIF1α promotes viral infections, inflammatory responses, and blocks type 1 IFN responses. Notably, HIF1α expression is higher in elderly patients, revealing another potential mechanism by which the elderly are predisposed to severe COVID-19 (88). Lastly, several comorbidities, including obesity and pulmonary hypertension, increase pre-existing HIF1α levels, which SARS-CoV-2 infection exacerbates, perhaps explaining the vulnerability of this patient demographic to severe COVID-19 (89).

Macrophages infected with SARS-CoV-2 reprogram their metabolism to become highly glycolytic, favoring SARS-CoV-2 replication and proinflammatory cytokine production (90). Monocytes accumulate lipid, which favors viral replication and proinflammatory cytokine production, and pharmacologically inhibiting DGAT1 to prevent lipid accumulation mitigates viral replication and cytokine production (91).

Regarding neutrophils, a recent study by Borella et al. investigated metabolic reprogramming in severe COVID-19 patients compared to healthy donors (92). In severe COVID-19 patients, HIF1α expression is higher in peripheral and BALF neutrophils, along with upregulated genes encoding proteins of glycolysis, glycogen metabolism, and gluconeogenesis and lower expression of genes of oxidative phosphorylation. As neutrophils depend on glucose metabolism for NETosis, glycolysis and glycogen stores are increased in neutrophils sampled from severe COVID-19 patients (92). Other than metabolic reprogramming due to direct SARS-CoV-2 virus infection, serum cytokines commonly elevated in severe COVID-19 also increase glycolysis in neutrophils to encourage NETosis (92).

A ketogenic diet can shift metabolism from glucose to ketones to reduce glycolytic capacity and mitigate SARS-CoV-2 pathophysiology. A randomized controlled trial (NCT04492228) is currently underway to evaluate a ketogenic diet as a path to alleviate COVID-19 (93). The role of HIF1α and metabolic reprogramming in predisposing vulnerable populations to severe disease still requires substantiation, but theoretically is likely.

### 3.2 Cytokine Storm of COVID-19

It became apparent early in the pandemic that, in its most severe form, COVID-19 is a systemic hyperinflammatory disease culminating in ARDS, multiorgan failure, sepsis, and ultimately death. Cytokine storm is an umbrella term describing a fatal endpoint of many inflammatory disorders, characterized by a systemic inflammatory response with constitutional symptoms and multiorgan failure. Initial studies in Wuhan, China showed elevated IL-1β, IL-7, IL-8, IL-9, IL-10, FGF, G-CSF, GM-CSF, IFN-γ, IP-10, MCP-1α and 1β, MIP-1, PDGF, TNF-α, and VEGF concentrations in COVID-19 compared to uninfected individuals. Furthermore, IL-2, IL-7, IL-10, G-CSF, IP-10, MIP-1α and1β, and TNF-α were higher in ICU COVID-19 patients than non-ICU patients (19). Future studies expanded these findings, demonstrating sustained increases in serum IL-2, IL-6, IL-10, and IFN-γ in severe COVID-19 cases, with higher IL-6 levels correlating with mortality risk. In addition, these cytokines reach their peaks at 4-6 days, in concert with the lowest T-cell count, and restoration of the T-cell lymphopenia correlates with decreases in IL-2, IL-6, IL-10, TNF-α, and IFN-γ levels (94). These immunological findings occur in conjunction with elevated inflammatory laboratory parameters, including higher alanine aminotransferase, lactate dehydrogenase, CRP, ferritin, and D-dimer, which correlate with disease severity (95).

The profiles of cytokine and non-cytokine mediators of severe COVID-19 have drawn resemblance to secondary hemophagocytic lymphohistiocytosis (sHLH) and macrophage activation syndrome (MAS) (96, 97). sHLH is a fatal complication of viral infections and sepsis, and MAS classically occurs in systemic idiopathic juvenile arthritis. The hallmark clinical and laboratory findings of sHLH and MAS are fever, cytopenias, hyperferritinemia, and pulmonary disease such as ARDS, occurring in ~50% of patients (97). Severe COVID-19 also features these abnormalities. For instance, hyperferritinemia of at least 4420 µg/L is a clinical cut-off value for MAS in bacterial sepsis and is a consistent feature of critically ill COVID-19 patients (81). The gene expression profile of activated lung macrophages in COVID-19 displays similarity with sHLH and MAS macrophages, and both sHLH/MAS and SARS-CoV-2 – induced cytokine storms include IL-2, IL-7, G-CSF, IP-10, MCP-1, MIP-1α and 1β, and TNF-α elevations (98). An NLRP3 inflammasome activation signature is a feature of COVID-19 and a marker of sHLH (99). Lastly, COVID-19 and HLH display marked NK cell exhaustion (46, 100).

However, despite these similarities, a recent study was the first to compare cytokine storms in COVID-19 and sHLH/MAS directly and found a clear distinction between the two (99). MAS cytokine storms exhibited higher IL-6, IL-18, IFN-γ, TNF-α, and CXCL9 than severe COVID-19, with IFN-γ, IL-18, and CXCL9 being significantly lower in COVID-19. In contrast, COVID-19 featured higher IL-5, IL-7, IL-17A, CXCL8, and VEGF; CXCL8 was three-fold higher in COVID than in MAS, and VEGF is absent in MAS-induced cytokine storms. In addition, although both COVID-19 and MAS feature hyperferritinemia and D-dimer elevations, their magnitude of increase in COVID-19 are markedly lower than in MAS (73). Lastly, studies showed no utility of using the HScore, the risk assessment protocol of HLH patients, to risk-stratify COVID-19 patients (101, 102), supporting the conceptualization of COVID-19 as a separate entity.

Comparative studies have also distinguished the cytokine storm of severe COVID-19 from bacterial sepsis, which displays monocytes lacking the ability to produce cytokines, and from severe influenza, which exhibits early interferon signatures, unlike severe COVID-19 (103). Furthermore, although widespread hypercoagulability characterizes severe COVID-19 and disseminated intravascular coagulation (DIC) secondary to sepsis, the coagulopathy pattern in these entities is distinct. Firstly, while sepsis-induced DIC is a fatal progression of ~30-40% of septic shock patients (104), only a few COVID-19 patients meet DIC criteria. Secondly, sepsis-induced DIC features thrombocytopenia, elevated parameters of the secondary coagulation cascade, and reduced antithrombin, whereas severe COVID-19 patients exhibit only minor reductions in these parameters but display higher fibrinogen and D-dimer levels (105). D-dimer elevations, as drastic as > 5 mg/L, despite anticoagulation, have been consistently elevated across several studies in ICU-admitted COVID-19 patients, with sudden increases suggestive of acute thrombotic events such as PE (106). Accordingly, D-dimer is considered a potential marker of severe disease, but objective cut-off points for risk stratification and therapeutic anticoagulation induction remain unidentified (107).

Elderly patients and those with comorbidities are most at risk of developing severe COVID-19. Indeed, adults > 65 years account for 80% of COVID-related hospitalizations and most deaths from COVID-19 (108). The predisposition of the elderly to severe COVID-19 has several underlying theories. However, to our knowledge, studies longitudinally comparing profiles of the hypercytokinemia in different age groups are few and far between. Regardless, there is some evidence that increasing age correlates with more dysregulated serum cytokine levels and inflammatory lab markers. In this regard, a study evaluating 44 adults hospitalized COVID-19 patients divided its cohort into two groups, young (<60 years) and old (>60 years), to measure differential immune responses to COVID-19 in different age groups. Older patients were hospitalized for longer, had a greater incidence of severe disease, and IL-27 elevation best correlated with age (109). A more recent study expanded on this by using the same age grouping to reveal higher serum IP-10, GM-CSF, IL-10, and TNF-related apoptosis-inducing ligand (TRAIL) but not IL-27 in older individuals, whereas younger individuals showed higher CCL5, a T-cell chemoattractant (110). The higher level of IP-10 and lower CCL5 expression in older patients suggest dysfunctional T cell responses, and IFN-γ elevations in older patients who succumbed to the disease are consistent with this suggestion. In contrast, increased levels of T cell-derived IL-2 IL-2, IL-7, IL-4, and IL-5, myeloid-derived IL-1α and 1β, and growth factors such as PDGF and TGF-α in younger individuals suggest that they develop more robust T cell responses in an attempt to clear the infection (110).

Older individuals are also more likely to develop the severe COVID-related dysregulated myeloid and lymphoid compartment phenotypes described in their respective sections. In addition, aging causes immunosenescence, in which naïve T-cell subsets decline. Additionally, aging creates a chronic low-grade sterile inflammatory state termed “inflammaging”; baseline proinflammatory cytokine levels in the lungs are higher in the elderly. These factors predispose older patients to dysregulated T-cell responses, which can cause disease progression. A more comprehensive review of aging-related changes in innate and adaptive immunity is comprehensively reviewed here (111).

However, cytokine storm may be an inaccurate descriptor of severe COVID-19 for several reasons. First, serum cytokine levels in critical COVID cases are insufficient to cause severe disease and are less robust than other cytokine storm-causing conditions (112–114). Second, histopathologic evidence distinguishes COVID-19 from other ARDS-causing viral illnesses by demonstrating predominant thrombosis and neoangiogenesis in COVID-19 lungs, with thrombi reported in the lung and several remote organs (23, 53). Third, despite thromboprophylaxis in severe COVID-19, clinical studies show a high incidence of thromboembolic complications. Lastly, studies have demonstrated COVID-19 pneumonia to be a slower disease than other respiratory viruses such as influenza A (115), and the duration of mechanical ventilation and ICU stay in COVID-19 patients is longer (5).

### 3.3 Endothelial Dysfunction in COVID-19: A Major Player in Pathogenesis

Endotheliitis may cause the angiocentric phenotype of COVID-19. An intact endothelium is crucial in maintaining hemostasis. Conversely, disruption of the endothelium exposes subendothelial collagen and tissue factors, precipitating a hypercoagulable state. In agreement with this, autopsy specimens reveal endothelial cell damage by detecting intracellular SARS-CoV-2 virus (23). Additionally, COVID-19 patients display higher markers of coagulopathy, including D-dimer, fibrinogen, prothrombin time (PT), and activated partial thromboplastin time (aPTT) (116, 117). In addition, a greater magnitude of D-dimer elevations is seen in COVID-19 patients when compared to other ICU-admitted patients (118), as well as other critical pneumonia cases not due to COVID-19 (119), with the incidence of venous thromboembolic complications in critically ill COVID patients far exceeding that of other ICU patients (120). Characterizing the molecular mechanisms participating in COVID-19 endothelitis could instigate the development and implementation of biomarkers to monitor and novel therapeutic targets to mitigate COVID-19. Below, we attempt to summarize the important mechanisms believed to be underlying COVID-19 endotheliitis, but for more detailed descriptions on this topic we refer readers to an exhaustive review by Smajda et al. (121).

#### 3.3.1 Evidence of SARS-CoV-2-Induced Endothelial Disease

This section lists some of the clinical data lending evidence to SARS-CoV-2-induced endotheliitis. A 3 µm thick glycocalyx protects the endothelium (eGC), estimated clinically by the perfused boundary region (PBR). Thinning of the glycocalyx, heralded by a higher PBR, occurs in COVID-19. Numerous investigators report eGC damage as manifested by elevations in PBR (122) and eGC constituents such as syndecan-1, elevated eGC-damaging heparanase-1, and lower heparinase-2, which inhibits heparinase-1 (122). Upregulation of vascular adhesion molecules on endothelial cells also signifies their activation. Indeed, P and E-selectin, ICAM-1, VCAM-1, and PECAM-1 level correlate positively with viral load, disease severity in a grade-dependent manner, and mortality (123–126).

Tie inhibition mediates the switch of cells from an anticoagulant to a procoagulant phenotype. Angiopoietin-1 activates Tie2 to signal vascular quiescence. Angiopoietin-2, released from activated endothelial cells, competitively inhibits Tie2 to induce a pro-adhesive, proinflammatory and hyperpermeable endothelial cell signature (127). Angiopoietin-2 levels are elevated in COVID-19 and correlate with SARS-CoV-2 load, serum D-dimer and CRP (128). Furthermore, angiopoietin-2 levels are higher in critically ill patients than hospitalized and outpatients and in non-survivors compared to survivors. Accordingly, angiopoietin-2 levels predict ICU admission and mortality reliably (129).

The vascular endothelial growth factor (VEGF) family, including VEGF A to E and PIGF, mediate angiogenesis and lymphangiogenesis. VEGF-A is elevated in COVID-19 compared to healthy controls and is higher in critical COVID-19 cases than non-critical cases. Soluble levels of Flt-1, a truncated form of the VEGF-A receptor, are also markedly elevated in COVID-19 patients and predict the need for mechanical ventilation, vasopressor support, and mortality (129). A prospective multicenter study showed VEGF-A to be one of the best predictors of COVID-19 disease severity (130), and another study attempting to associate VEGF family members with in-hospital mortality due to COVID-19 reported that VEGF-A, PIGF, and FGF-2 significantly increased with disease severity (p < 0.001) with a serum PIGF cut-off of above 30pg/mL the best predictor of in-hospital mortality on survival analysis (p = 0.001). Hypoxia is a well-known trigger of VEGF, and autopsy findings of COVID-19 lung disease show hypoxia-related gene expression of HIF1α and an increase in intussusceptive angiogenesis, which correlates with serum angiogenesis markers including VEGF (23). The roles of VEGF and angiogenesis in COVID-19 and ARDS are discussed in detail here (121, 131–133).

Lastly, von Willebrand Factor (vWF) is synthesized by endothelial cells, stored in Weibel-Palade bodies, and released during endothelial activation by proinflammatory stimuli. vWF is essential for platelet aggregation and plays a significant role in thrombosis. Therefore, vWF constitutes a link between inflammation and thrombosis. Numerous studies show that vWF is elevated in COVID-19 (134) and is higher in critically ill patients (135). Furthermore, independent cohorts have yielded similar results that vWF levels on hospital admission predict mortality, with higher vWF levels in non-survivors (126, 136, 137). In addition, A Disintegrin And Metalloproteinase with ThromboSpondin motifs 13 (ADAMTS13), which cleaves large multimeric vWF polymers into monomers to prevent thrombosis, is reportedly depleted in mechanically ventilated COVID-19 patients, and an eightfold higher vWF-to-ADAMTS13 ratio occurs in ICU-admitted and mechanically-ventilated COVID-19 patients (137, 138).

#### 3.3.2 Role of NETs, Platelets, and Complement in Endotheliitis

Numerous studies have proposed that endothelial cell activation may result from NETs production from neutrophils. NET markers, including cell-free DNA (cfDNA), histones (cit-H3), and myeloperoxidase (MPO), are elevated in COVID-19 and correlate with disease severity (49), serum of individuals infected with SARS-CoV-2 can induce NETosis (139, 140), and *in vitro* studies have shown SARS-CoV-2 directly infecting neutrophils and inducing NETosis (141, 142). Furthermore, NETs occur within pulmonary, renal (143), and cardiac (144) microthrombi in COVID patients. These findings informed suggestions that perhaps NET markers constitute reliable prognostic biomarkers (145–147). Since NETs are highly procoagulant, they contribute to the immunothrombosis and pulmonary autopsies of COVID-19 consistently reveal NETs in close association with damaged alveoli, as well as NET-platelet complexes (148). Accordingly, administering NET-lysing DNase abrogates IL-6 and TNF productions and reduces the BALF-plasma cytokine gradient of these cytokines (149).

Platelets are activated during COVID-19 and bind neutrophils through integrins and induce NETosis through various platelet-derived particles, such as HMGB1 (150, 151), although other platelet-derived compounds may be involved (148). Alternatively, NETs-induced endothelial damage results in Von-Willebrand factor (vWF) exposure, which binds platelets and activates them. These NETs could be released directly by viral neutrophil infection or indirectly *via* IL-1β. Therefore, this neutrophil-platelet circuitry promotes NETosis in COVID-19, rationalizing its importance as a potential therapeutic target (148).

Investigations into potential complement dysregulation in COVID-19 stemmed from such observations in the original SARS infection (152, 153). Immunohistochemical analysis of COVID-19 lung autopsies reveals high expression of the complement components MBL, C4, C3, and C5b-9 in alveolar epithelial cells (154), as well as in COVID-induced acute kidney failure. Moreover, higher serum markers of complement activation occur in hospitalized COVID-19 patients than hospitalized patients with a non-COVID respiratory disease, and in patients who required mechanical ventilation compared to those who did not (155, 156). Remission is accordingly associated with a decline in complement markers.

The SARS-CoV-2 S protein activates the alternative pathway *in vitro* (157). Additionally, the SARS-CoV-2 N protein binds MASP-2 leading to complement hyperactivation – *via* the mannan-binding lectin (MBL) pathway – and aggravated lung injury (152, 158). Since complement dysregulation perpetuates inflammation, which in itself is a prerequisite for NETosis of infiltrating neutrophils, a potential circuitry between complement-mediated cytokine storms and NETosis might exist (148). Interestingly, a recent study showed the accumulation of CD16+ T cells in severe COVID-19, which degranulate to release neutrophil and monocyte chemoattractants. Furthermore, C3a induced this T cell phenotype in severe disease, and C3a and CD16+ T cell responses correlated with COVID-19 severity and outcome (159). Therefore, the depth of the interplay between various components of the innate response and platelets is profound. Further characterization of these processes and correlating future findings with disease severity is necessary to rationalize using these factors as biomarkers and potential therapeutic targets.

## 4 Adaptive Immune Response Against SARS-CoV-2

The adaptive immune response constitutes the central host defense system against viruses and is crucial in clearing SARS-CoV-2 infection. As described above, SARS-CoV-2 initially replicates in the nasopharyngeal passageways, which clinically manifests as asymptomatic infection or with mild flu-like symptoms. The eventual extension of infection into the lungs can create a disease severe enough to require hospitalization. However, effective initial innate responses can mitigate this by creating an antiviral state in the tissues *via* type I and III IFN responses and by triggering adaptive immune responses (41). The adaptive immune response comprises B cells, which differentiate into plasmablasts producing specific antibodies, CD4+ helper-T (Th) cells that possess numerous helper functions, including B cell help for the production of high-affinity antibodies, and augmentation of innate responses *via* IFN-γ and by stimulation of CD8+ T cells (CTLs), which kill infected cells. Furthermore, CD4+ Th cells, in addition to the functions above, also exert direct Th1-mediated antiviral activity to mitigate SARS-CoV-1 infection in mouse models (160).

### 4.1 What Does Protective Immunity Against SARS-CoV-2 Constitute?

As discussed above, SARS-CoV-2 possesses a central immune evasion mechanism of inhibiting IFN responses (103, 161), which impairs the development of an effective innate immune response, delaying stimulation of the adaptive response until after SARS-CoV-2 has established significant lung disease.

Therefore, the likelihood of effective host defense against SARS-CoV-2 or the development of clinically significant COVID-19 depends on the timeliness of an adaptive immune response. Early stimulation of adaptive, particularly T cell, defense resolves infection in asymptomatic or mild disease, whereas delayed T cell responses are non-homeostatic and can worsen COVID-19 by amplifying the innate response (160). Numerous clinical studies have shown this to be the case (162, 163). In the latter scenario, the inhibition of IFN responses plays a crucial role. However, other factors such as age, which is associated with a decline in naïve CD4+ Th cell counts, and HLA restrictions, which influence the T cell repertoire developed in response to infection, also influence COVID-19 trajectories.

This section discusses the seminal studies informing our current understanding of adaptive immune responses against SARS-CoV-2. Pertinent clinical questions early in the pandemic that guided the initial studies pertained to what immunophenotype characterized mildly affected convalescent COVID-19 patients versus how this phenotype changed in severe COVID-19 patients. Furthermore, elucidating the immunodominant epitopes of SARS-CoV-2 targeted by the T cell arm of the adaptive response was imperative for predicting the efficacy of the formerly developing vaccines, all of which utilized the S protein as the immunogen. Lastly, assessing how long immunologic memory persisted after infection or vaccination was essential for developing vaccines.

The following discussion concentrates on T cell responses rather than humoral immunity, as the data suggest a dispensable role of antibody responses in clearing SARS-CoV-2 infection and alleviating disease severity. Antibodies are more effective in conferring a sterilizing immunity characterized by extracellular neutralization of virions before infection occurs, but T cells are responsible for clearing the infection. To this end, patients with X-linked agammaglobulinemia recover from COVID-19 without severe symptoms (164), patients on B-cell depletion therapies recover from COVID-19 without complications (165), and other instances where neutralizing antibody responses were suboptimal but T cell responses were preserved (166).

#### 4.1.1 Immunological Signatures in Convalescent and Acute COVID-19

Studies investigating the immunophenotype of convalescent COVID-19 patients versus acute COVID-19 patients highlight the importance of T cell immunity.

Grifoni et al. investigated the targets of the T cell response against SARS-CoV-2 in convalescent COVID-19 patient sera (167). Robust CD4+ Th and CD8+ CTL responses occurred in 100% and 70% patients, respectively, with most but not all reactions against the S protein, which was encouraging for prior developing vaccines (167).

Moderbacher et al. evaluated these same parameters in acute COVID-19 cases and correlated the phenotype of each subset of adaptive immunity (antibodies, CD4+ Th, and CD8+ CTLs) with disease severity (162). In contrast to the uncomplicated convalescent COVID, acute disease featured much more variable CD4+ T cell responses in 77% of acute COVID-19 patients than 100% in convalescents, and 27% of CD4+ responses were designated weak (162). Further, correlation with disease severity revealed that the CD4+ response best predicted disease severity, not the antibody response, suggesting that the T cell arm of adaptive immunity is critical in clearing SARS-CoV-2 after infection.

Early SARS-CoV-2-specific CD4+ Th cell responses are associated with mild disease, whereas later induction – as late as 22 days post-symptom onset (PSO) – is associated with severe disease (162). Additionally, this study also observed SARS-CoV-2–specific follicular helper T cells (Tfh) in acute cases, with their frequency inversely correlating with disease severity. Notably, increasing age correlates with disease severity, with the adaptive response appearing more uncoordinated in older individuals (162). Age is indeed associated with a decline in naive CD4+ and CD8+ T cell populations, termed immunosenescence (168). The CD8+ CTL compartment is particularly affected, impairing SARS-CoV-2 clearance and increasing the likelihood of developing consequent immunopathology.

Although representing a minority of symptomatic COVID-19 cases and deaths, children and adolescents can suffer from a rare complication of COVID-19 called the multisystem inflammatory syndrome (MIS-C), which peculiarly manifests weeks after the infection and resembles a Kawasaki disease-like illness (169). The pathogenesis of MIS-C involves hyperinflammation similar to COVID-19, as evidenced by treatment with immunomodulating therapies such as IVIG and steroids (170). However, the types of cytokine storms induced by COVID-19, MIS-C, and Kawasaki disease are distinct. Kawasaki's disease characteristically features IL-17 elevations and vasculitis-related markers, whereas MIS-C does not. Alternatively, MIS-C may involve autoantibodies formed against casein kinases (171). Profiling the adaptive response in MIS-C reveals profound T cell lymphopenia with an exhausted phenotype and persistent B-cell plasmablast response, which is higher than in severe COVID-19 (172). Additionally, MIS-C features a preferential activation of CX3CR1+ CD8+ T cells, although their proportion is unchanged compared to COVID-19, which correlated with D-dimer elevations, thrombocytopenia, and ICU admissions (172). However, the mechanism underlying this preferential activation remains unidentified.

#### 4.1.2 Immunologic Memory to SARS-CoV-2

Studies have determined the length of protective memory responses induced by SARS-CoV-2 infection and vaccination. Early longitudinal analyses following patients infected with SARS-CoV-2 revealed a lower incidence of reinfection, suggesting the existence of working SARS-CoV-2-specific memory (173, 174).

A landmark study by Dan et al. longitudinally characterized immune profiles in previously infected individuals up to 8 months post-infection (175). Neutralizing antibody titers decline modestly over 8 months (175), and ~25% of patients become seronegative over 6 months (176). T cell responses are more durable than antibodies, with memory CD4+ and CD8+ responses detected in ~90% and 70% of individuals (175, 177), respectively. Memory CD8+ responses take up an effector phenotype while circulating memory CCR6^+^ Tfh cells, which accounted for most circulating CD4+ responses, increased over the eight months and were associated with reduced COVID-19 severity (175).

Although neutralizing antibody titers decay relatively more rapidly than T cell responses, SARS-CoV-2-specific memory B cell responses are very stable, detected in 100% of subjects 8 months post-infection (175). Furthermore, the frequencies of SARS-CoV-2-specific memory B cells increased over time, higher at 6-month follow-up than 1 month PSO (175); this is consistent with findings in other independent patient cohorts (178). Almost all memory B cells were spike-specific IgG (95%), with only 5% IgA responses. Furthermore, memory B-cells continue to undergo affinity maturation due to continued germinal center responses after SARS-CoV-2 infection (175). However, antibody titers were not correlated with SARS-CoV-2-specific CD4+ T cells (175), making large-scale detection and monitoring of T-cell responses more cumbersome, as in many instances, antibody responses are a surrogate marker of T cell activity. The striking feature of this immunologic memory response is its heterogeneity, but what underlies it is unknown, with studies implicating HLA polymorphisms, aging, and other factors (160).

Recent studies have also demonstrated differences in vaccination-induced immune responses between naïve individuals and those who recovered from COVID-19. Early after vaccination, individuals who recovered from COVID-19 develop more robust humoral responses than naïve subjects, whereas naïve subjects develop better cell-mediated reactions (179). After immunization (7-8 months), neutralizing antibody titers drop to comparable levels in both groups, and similar observations apply for T cell responses (179). However, some studies demonstrate higher antibody titers in previously infected patients early and later after vaccination (180, 181). Lastly, comparative studies on the differential post-vaccination immune profiles of naïve subjects and individuals previously infected with specific SARS-CoV-2 VOCs have not been conducted.

These studies measured adaptive memory responses in peripheral blood, but adaptive immunity exerts its protective effect at the level of the tissues. Therefore, more recent efforts focus on characterizing immunologic memory at the level of the tissues. To this end, immune profiles in the lungs, spleen, bone marrow, and lymph nodes (LNs) of COVID-positive organ donors up to 6 months post-infection reveal memory SARS-CoV-2-specific T and B cells, including potent Tfh and germinal center responses, particularly in the lung and its draining LNs (182). In addition, the abundance of memory T and B-cells in the lungs and LNs correlated with the magnitude of SARS-CoV-2-specific memory cells in the circulation (182). Therefore, recent efforts on the tissue-level immune memory elicited upon SARS-CoV-2 infection have yielded encouraging results. However, such responses after vaccination also need to be profiled.

#### 4.1.3 Immune Profiles in Long COVID-19

Individuals who recover from severe COVID-19 can chronically experience severe pulmonary and extrapulmonary symptoms, collectively termed PACS or long COVID. Multiple prospective cohort analyses have shown significant morbidity, mortality, and health expenditures in individuals suffering from PACS (183, 184). On imaging evaluation, these changes are predominantly due to extensive lung fibrosis post-COVID-19 (185).

Recently, a study profiled the adaptive response in the BALF and blood of aged (> 60 years old) COVID-19 convalescents suffering from long COVID compared to healthy aged controls. Initial CT and pulmonary function tests (PFTs) in the aged convalescents revealed lung fibrosis and PFTs, suggestive of restrictive lung disease (186).

High-dimensional flow cytometry of the blood and BALF revealed differential immune profiles: in the COVID-19 convalescent cohort, frequencies of γδ T cells, B cells, and particularly CD8+ T cells within BALF increase. In addition, B cells and CD4+ T cells signatures display a tissue residency phenotype, with increased RBD-specific memory B cells and a CD4+ T cell-dependent elaboration of SARS-CoV-2-specific IgG antibodies (186). Importantly, BAL CD8+ in convalescents take up a T_RM_ (both CD69^+^CD103^+^ and CD69^+^CD103^-^) signature and elaborate higher percentages of IFN-γ and TNF cytokines upon stimulation compared to their counterparts in the peripheral blood. Furthermore, CD69^+^CD103^-^ T_RM_ CD8+ T cells have shown to correlate with PFT results negatively but positively correlate with pathologic PFT and CT findings, which is consistent with murine models of influenza virus infection, which demonstrate that depleting CD8+ T cells in the respiratory tract alleviates lung disease post-influenza viral pneumonia (186, 187).

ScRNA-seq reveals extensive, clonally expanded T cells in the BALF and the circulation of convalescent COVID-19 patients. BALF CD8+ clusters in COVID-19 convalescents display a tissue residency transcriptomic signature involving upregulated genes of myeloid cell inflammation, including Lyz, S100A8, and S100A9 (186). CD69^+^CD103^-^ T_RM_ CD8+ T cells have an activated phenotype and express higher levels of NKG7, a cytotoxic proinflammatory molecule, and granzyme K, a proinflammatory and profibrotic granzyme. Lastly, CXCR6^+^ CD8^+^ T cells accumulate in the lungs of convalescents compared to healthy controls, with their presence positively correlating with fibrotic lung changes on PFTs and imaging. The gene expression signature of these CXCR6^+^ CD8^+^ T cells approximates those of tissue-damaging CXCR6^+^ CD8^+^ T cells in nonalcoholic steatohepatitis (186, 188).

Therefore, accumulating CD69^+^CD103^-^ and CXCR6^+^ CD8+ T cells elaborate proinflammatory and profibrotic cytokines to cause lung fibrosis in PACS.

#### 4.1.4 Pre-Existing T-Cell Responses and Cross-Reactivity

Intriguingly, the previous study by Grifoni et al. (167) and numerous other reports (163, 189–192) show that many unexposed controls – who had their blood samples collected before the COVID-19 pandemic – test positive for SARS-CoV-2 cross-reactive T cells. The relevance of cross-reactive humoral immunity against SARS-CoV-2 in unexposed individuals is minimal in comparison (193). To this end, Mateus et al. identified 142 epitopes recognized by these cross-reactive CD4+ memory T cells and demonstrated significant sequence homology between these epitopes and peptides of human common cold coronaviruses (HCoVs) – OC43, 229E, NL63, and HKU1 (194). Subsequently, peptide pools composed of more than 100 HCoV peptide homologs were reacted with these CD4+ memory T cells from unexposed individuals, revealing much greater reactivity to these homologs than to SARS-CoV-2 peptides. Lastly, the researchers created *in vitro* T cell lines using these epitopes, which recognized specific HCoV peptides even better than SARS-CoV-2 epitopes (194). Therefore, prior exposure to HCoVs explains the pre-existing memory CD4+ T cell immunity. However, prior exposure to HCoVs alone cannot account for all pre-existing T cell cross-reactivity (195), because unexposed individuals also exhibit noncognate cross-reactivity (i.e., not explainable by prior exposure to HCoVs). To this end, another study showed that pre-existing T cell responses to SARS-CoV-2 epitopes derive from previous exposure to common viral antigens such as Influenza and CMV but not the HCoVs (196). Nevertheless, the significance of these cross-reactive T cells in contributing to COVID-19 outcomes and responses to vaccination remains an area of active investigation.

Bacher et al. revealed the pre-existing CD4+ T cell memory response as displaying only low functional avidity and an increase in their proportion in the CD4+ T cell compartment with aging (197). Furthermore, comparing TCR avidities of these memory T cells in hospitalized COVID-19 patients versus mild COVID-19 revealed their expansion in severe cases. In severe COVID-19, the TCR repertoire was also broader and of low avidity, similar to the pre-existing memory CD4+ response. In contrast, a highly clonally expanded and cytotoxic Th1 response characterizes mild COVID-19. Therefore, pre-existing T-cell reactivity may contribute to low avidity and polyclonal responses in severe COVID-19, which would explain the association of COVID-19 with aging, as the proportion of these cells increases with aging. However, the presence and contribution of pre-existing CD4+ memory T cells in these hospitalized patients was unknown, but similar T-cell responses profiles in mildly affected patients were associated with pre-existing T-cell immunity (197).

Contrastingly, recent studies have suggested a beneficial role of pre-existing memory T cell cross-reactivity in SARS-CoV-2 infection and vaccination. In this regard, Loyal et al. demonstrated recruitment of pre-existing SARS-CoV-2 cross-reactive T cells into the immune response upon SARS-CoV-2 infection (198). Furthermore, the magnitude of this cross-reactive T cell response was associated with higher neutralizing antibody titers. In agreement with this, many studies have reported that prior infection by HCoVs may be associated with less severe COVID-19.

Furthermore, significant T cell responses against HCoVs are evident in unexposed individuals, with a subset of this response accounted for by high avidity CD4+ T cells (199). A decrease in the high avidity cross-reactive CD4+ response with aging was observed and could explain the vulnerability of the elderly population to severe COVID-19 (198). Lastly, the immune response after Pfizer-BioNTech (BNT162b2) vaccination superseded even that seen after natural SARS-CoV-2 infection in terms of S protein-specific T cell and neutralizing antibody responses. Importantly, immune responses against regions of the S protein sharing homology with HCoV peptides exhibited secondary immune response kinetics, while other regions did not. Thus, perhaps pre-existing memory T cell cross-reactivity accounts for the rapid protection provided by the BNT162b2 vaccine and the requirement of booster doses in the elderly (198). In agreement with this, a recent study by Mateus et al. investigated the adaptive response elicited by a low 25µg dose of the mRNA-1273 Moderna vaccine and showed that pre-existing T cell responses enhanced antibody responses, S protein-specific Tfh cells, and total CD4+ T cell count (200). In addition, cross-reactive memory T cells correlate with higher neutralizing antibody titers six months after vaccine administration (200).

Collectively, therefore, the pre-existence of SARS-CoV-2 cross-reactive T cells – the majority but not all of which originate from prior exposure to HCoVs – enhances the immune responses against SARS-CoV-2 infection and vaccination. However, increasing age and possibly other unknown factors may, in turn, cause these cells to drive immunopathological low avidity and polyclonal T cell responses in severe COVID-19 cases. However, although pre-existing cross-reactive T cells influence adaptive immune responses, their role in protecting against SARS-CoV-2 infection is likely minimal. Indeed, SARS-CoV-2-induced CD4+ responses targeted 280 epitopes, 227 of which were not seen in unexposed donors, indicating that infection generates a new TCR repertoire (201). However, an interesting study showed that T cell subsets in close contacts of symptomatic COVID patients showed that individuals who possess cross-reactive memory T cells remain PCR-negative despite frequent exposure to COVID-19 patients (202), suggesting that cross-reactive memory T cells protect against SARS-CoV-2 infection.

#### 4.1.5 SARS-CoV-2 T Cell Epitopes

An exhaustive review by Grifoni et al. enumerated the total number of T cell epitopes discovered by numerous studies and found 1052 non-redundant class I epitopes and 352 class II epitopes (203). Most studies show the S, M, and N proteins to be the primary, i.e., the immunodominant, targets of CD4+ and CD8+ T cell responses. In addition, strong responses against ORF3, ORF8, ORF1ab (nsp 13), and nsp3, 4, 6, and 12 are also present (167, 201, 204). The antigen’s size and expression level determine the number of epitopes on an antigen and, therefore, the robustness of the T cell response against that antigen. For instance, the SARS-CoV-2 structural proteins S, M, and N are highly expressed and are also the most immunodominant (167).

Studies also identified the most immunodominant regions of the antigens mentioned above. For example, the spike RBD was not an immunodominant target of CD4+ responses; discrete areas adjacent to the RBD, such as residues 154-254, 296-370, and 682-925, were more immunodominant for CD4. In comparison, the S protein immunodominance pattern across an antigen for CD8 is more homogenous throughout the protein. This same pattern is maintained for M and N proteins and is more exaggerated in the case of nsp3 and nsp12 (201, 203). Thus, in summary, while CD4 responses focus on discrete regions of the above-mentioned immunodominant antigens, CD8 epitopes are more homogenously distributed throughout the protein.

The seminal study by Tarke et al. showed that, for CD4+ T cells, the most immunodominant epitopes are promiscuous (i.e., recognized in at least three donors), and each individual recognizes 15-20 class I and class II epitopes (201). Furthermore, because of HLA restrictions, each individual recognizes and generates responses against different epitopes, which produces a great diversity of T cell responses between individuals. This study also demonstrated HLA restrictions for 178 out of 280 different epitopes for CD4+ T cell responses (201).

Other studies expanded these findings, and the review by Grifoni et al. identified a total of 1,191 class I restrictions and 783 class II restrictions in the literature (203). The median epitopes per HLA allele is 35 for class I and 12 for class II. The most common class I restrictions are associated with A*02:01, A*24:02, A*01:01, and B*07:02 allelic specificities, and the most common class II restrictions occur in the context of DRB1*07:01 and DRB1*15:01 (203). Lastly, although average individuals target between 15-20 class I and II epitopes, the response efficacy may vary as manifested in the significant variability of COVID-19 in disease severity (201). Gittelman et al. (205), Snyder et al. (206), Shomuradova et al. (207), and Gangaev et al. (204) associated certain TCR with the recognition of specific epitopes, and Snyder also developed a methodology to diagnose SARS-CoV-2 infection based on TCR sequencing (206). Therefore, future studies expanding these findings could provide mechanistic insights into SARS-CoV-2 pathogenesis and have diagnostic and therapeutic implications (203).

SARS-CoV-2 variants acquire mutations in the S protein but conserve the other epitopes that generate robust T cell immunity. Studies comparing antibody and T cell responses generated against VOCs in COVID-19 convalescents and recipients of the Moderna and Pfizer-BioNTech vaccines showed variable degrees of impairment in antibody responses, but the preservation of CD4+ and CD8+ T cell responses (208, 209). Indeed, only 7% and 3%, respectively, of CD4+ and CD8+ epitopes are affected by mutations in VOCs (208). Therefore, vaccine-induced T cell responses against the SARS-CoV-2 variants appear to be a promising vaccine target to prevent severe infection.

Recent studies show similar findings regarding the Omicron variant. Although Omicron features in a more significant percentage of mutations in S-specific epitopes targeted by vaccine-induced CD4+ Th (14%) cells and CD8+ CTLs (28%) than the previous variants, a large amount (86% and 72%, respectively) of the CD4+ and CD8+ S-specific response are conserved (210). Another recent study utilized the 280 CD4 epitopes identified by Tarke et al. in Omicron to determine their degree of conservation. 80.4% (74/92) and 94.7% (178/188) of spike and non-spike epitopes, respectively, were conserved in Omicron, and 90.2% (252/280) of the aforementioned CD4+ T cell epitopes were completely conserved (211). As for the 454 class I-restricted CD8 epitopes, 88.4% (137/155) and 98.3% (294/299) of spike and non-spike epitopes, respectively, were conserved, and 94.9% (431/454) of the CD8 epitopes were conserved in Omicron (211). Therefore, although the VOCs, including the recent Omicron variant, evade humoral responses to a considerable degree (212–214), T cell epitopes and responses are primarily conserved (211), which is encouraging for the, which is encouraging for the COVID-19 vaccines currently in use.

To conclude this section, the T-cell arm of adaptive immunity is more critical than antibody responses in clearing SARS-CoV-2 and reducing disease severity, although a coordinated adaptive immunity is the best correlate of protection. Furthermore, T cells show favorable evidence regarding clearing the already circulating and the inevitable future emerging SARS-CoV-2 variants, which can escape neutralizing antibody responses considerably. However, as discussed above, in the context of immunologic memory, the adaptive response to SARS-CoV-2 infection is incredibly heterogeneous, and antibody responses cannot predict T cell responses reliably. Consequently, there is a concerted effort to develop assays to measure T cell responses, such as the interferon-γ release assays (IGRAs) used in tuberculosis infection and vaccination (215).

### 4.2 T Cell Responses in COVID-19

#### 4.2.1 Lymphopenia

Lymphopenia affecting all T cell classes, particularly the CD8+ population, are hallmarks of severe COVID-19 (216, 217). The lymphopenia specifically affects central memory and naïve CTLs, which correlates with disease severity, whereas all Th subclasses are affected (217, 218). Contrastingly, neutrophil counts progressively increase with disease severity as discussed in neutrophil responses to COVID-19, and non-survivors show elevated neutrophil counts compared to survivors. Neutrophilia and T-cell lymphopenia manifest as an increased neutrophil to lymphocyte ratio. Indeed, the neutrophil-to-lymphocyte ratio is considered an independent predictor of COVID-19 severity (94, 219, 220). T-cell function and activation indices are also reduced, including TCRs, T cell surface markers, T cell migratory stimulators, and TCR signaling kinases, further hinting at a global impairment in T cell functionality (218, 221). Detectable protective T cell-mediated antiviral responses occur in mildly affected patients, and T cell recovery occurs in convalescent sera, reaching comparable levels to mild disease (94, 217).

Augmented extravasation into the interstitium of the lung and affected organs could explain lymphopenia. However, while post-mortem analyses reveal pulmonary lymphocytic infiltrates, these are not drastic enough to cause systemic lymphopenia. Interestingly, SARS-CoV-2 can directly deplete T cells. In this context, a preprint study conducting postmortem examinations of the spleen and lymph nodes reports elevated markers of T cell apoptosis and FAS expression (10). Flow cytometry studies have also shown similar findings, implicating activation-induced cell death (AICD) as the causative mechanism (222). Alternatively, IL-2 – IL-2 receptor signaling defects also contribute to T cell loss in critical patients (223). From the above data, the mechanism underlying SARS-CoV-2 lymphopenia remains unproven but may involve multiple pathways.

#### 4.2.2 Severity-Dependent T Cell Phenotypes in COVID-19

CD4+ Th, CD8+ CTL, and NK cell exhaustion – defined as the upregulated and sustained expression of cell surface immune checkpoint inhibitors – occurs in severe COVID cases (224, 225). T cell exhaustion features an increased expression of programmed cell death protein 1 (PD1) and T-cell immunoglobulin mucin 3 (TIM-3), and the extent of CD4+ Th cell exhaustion is more significant in patients admitted to ICUs versus those with milder disease (42, 226–228). CD8+ cytotoxic T cell and NK cell exhaustion are also marked in severe cases, as evidenced by the increased expression of surface NKG2A, which correlates with the numerical depletion of these cells and disease progression (95). High circulating IL-6 increases NGK2A expression on CTLs and NK cells, as shown previously in influenza (229, 230). Consistently, in convalescing and recovering patients, CTL and NK cell counts normalize with decreased expression of NKG2A (45, 204). Based on the reduced effector functions of CD8+ CTLs in COVID-19, studies have suggested that CD8+ T cells – unlike CD4+ Th cells – are immunoprotective in COVID-19 (204, 231).

Contradictory reports exist on the CD8+ T cell phenotypes in severe COVID-19. Studies report either robust activation of the T-cells in critical cases (232) with a high percentage of IFN-γ-producing CTL cells (233) or severe COVID-19 as an immunosuppressive phenotype, exemplified by profound declines in IFNγ- and TNF-producing T cells (225). Therefore, whether the exhausted CD8+ T cell phenotype is primarily due to exhaustion or secondary to hyperactivation remains unclear. To this end, a recent study compared the immune landscape in 11 postmortem COVID-19 lungs versus three non-COVID-19 lungs (234). An immunosuppressive phenotype was detected, as evidenced by greater TIM-3 and PD-1 in COVID-19 lungs. The immunosuppression also selectively affected T-cells. Men exhibit greater magnitudes of immunosuppression than women, and a positive correlation between TIM-3 expression and aging exists in men but not women (234), which perhaps contributes to males being more vulnerable to severe disease than females.

The apparent critical role of PD-1 and TIM-3 as immunosuppressive mediators rationalizes their inhibition to mitigate critical COVID. However, blocking immune checkpoints may conversely augment dysfunctional T-cell responses in severe patients and, in turn, mediate immunopathology. Indeed, cancer patients receiving these therapies develop severe COVID-19, but whether checkpoint inhibiting therapies or the generally increased vulnerability of cancer patients to severe infections explain this remains unknown (234). Therefore, more extensive studies on patients not suffering from comorbidities, which independently increase the likelihood of severe disease, are needed.

#### 4.2.3 Mechanisms Underlying T Cell Impairment in COVID-19

A recent study proposed a model where SARS-CoV-2 initially infects resident alveolar macrophages. These, in turn, produce chemokines that attract cross-reactive memory T cells, which produce IFN- γ to activate these macrophages (5). A central factor in this scenario is cross-reactive memory T cells, which several studies have confirmed in the elderly and severe COVID patients. Furthermore, cross-reactive T cells display reduced antiviral responses secondary to stimulation with SARS-CoV-2 peptides compared to patients who recovered from COVID-19. The authors further stated that these ineffective cross-reactive T cells possibly explain the increased susceptibility of such demographics to severe COVID infection (5). However, these findings remain controversial due to the evidence on the beneficial role of pre-existing cross-reactive T cells.

An interesting preprint study generated SARS-CoV-2 reactive CD4+ T cells from healthy donors to compare the anti-S and anti-M protein CD4+ T cell responses (235). While the anti-S protein CD4+ T cell responses approximated conventional CD4+ signatures to other viral antigens such as CMV, the M-specific CD4+ T cell lines showed a distinct transcriptional signature featuring suppression of interferon signaling, not dissimilar to findings in severe COVID-19 patients (235). Therefore, perhaps severe SARS-CoV-2 infection seen in specific individuals is because of an imbalance of CD4+ T cells in favor of M-specific T cell lines that drive pathologic responses (235). It will be interesting to see how future studies attempt to uncover the driving factors of specific T cell signatures in varying severities of COVID-19; advancing age associated with T cell immunosenescence and HLA restrictions are likely candidates.

Recent data suggest an effect of SARS-CoV-2 on the homing behavior of T cells through altering chemokine receptor patterns (218). Indeed, BALF analysis of mild-to-moderate COVID demonstrates a highly clonally expanded CD8+ T cell infiltration, whereas severe cases show more heterogeneously expanded CTLs (52). Additionally, the immunodominant epitope – i.e., the epitope representing the predominant target of T-cell immunity – is variable in SARS-CoV-2, with robust immune responses against the N protein, S protein, and M protein against specific ORFs (201, 236). This broad TCR repertoire in COVID-19 starkly contrasts with the original SARS-CoV-2 infection, where the S protein is the primary target of host responses. This apparent lack of an immunodominant epitope may be due to aberrant antigen processing, which could, in turn, impair T cell reactions (237). SARS-CoV-2 specific CD4+ Th memory cells retrieved from severely afflicted patients respond to a wider variety of epitopes than in milder cases (52, 237). However, the reason behind why such a diverse and robust antiviral response cannot eradicate SARS-CoV-2 remains unanswered.

Future studies should explain why a hyperactivated T cell response cannot eradicate SARS-CoV-2. In addition, why severe illness disproportionately occurs in specific demographics remains a contentious issue. Studies investigating these highly pertinent questions would further our understanding of some critical, clinically relevant problems and improve the identification of at-risk individuals.

### 4.3 B cell and Humoral Responses in COVID-19

Humoral antibody responses constitute an essential part of adaptive immunity against viruses, including SARS-CoV-2 (238). For example, antibodies against the RBD of SARS-CoV-2 sterically hinder virus-host cell interactions, neutralizing virus infectivity. Alternatively, IgG antibodies binding viral surfaces promote opsonization, i.e., phagocytosis by macrophages. Antibodies also directly induce the clearance of virus-infected cells through NK cell activation by antibody-dependent cellular cytotoxicity (ADCC). Lastly, memory B cells are crucial to long-lasting immunity against viruses (239).

B cells are classified based on surface CD27 and CD38 expression into transitional (CD27^-^CD38^++^), naïve (CD27^-^CD38^+^), plasmablasts (CD27^++^CD38^+^), memory unswitched (IgD^+^CD27^+^CD38^-^), memory switched (IgD^-^CD27^+^CD38^-^), and double-negative cells (CD27^-^CD38^-^) (240). The prototypical B cell response to a viral infection features an initial extrafollicular (EF) B cell signature, which involves the differentiation of naive B cells into plasmablasts that generate low-affinity and temporary antibodies. Simultaneously, viral infection also elicits recall of pre-existing cross-reactive memory B-cells. Some B cells migrate to germinal centers (GC) where they undergo somatic hypermutations (SHM) to generate long-lived plasma cells, which continuously produce high-affinity antibodies and class-switched memory B cells, which produce high-affinity antibodies upon antigenic re-exposure.

SARS-CoV-2 infection generates potent neutralizing antibody responses against the SARS-CoV-2 N and S proteins, and most individuals seroconvert ~10 days post-symptom onset (PSO). The RBD of S-protein is targeted by 90% of nAbs, with variable degrees of neutralizing activity against the NTD. Furthermore, this highly convergent SARS-CoV-2 neutralizing antibody response against the RBD of S and therapeutic monoclonal antibodies targeting these same sites exert tremendous selection pressure for SARS-CoV-2 to develop mutations in these sequences (241). Indeed, the already present VOCs harbor common mutations in sequences recognized by these neutralizing antibodies and mAbs, resulting in reduced efficacy of neutralizing antibody responses and therapeutic antibody cocktails (212, 241–244).

These early antibodies derive from naïve B-cell-derived extrafollicular responses, as studies show that these IgG nAbs possess little to no SHM (245, 246) and appear in the blood at the same time as plasmablasts (42). Indeed, B-cell responses in mild SARS-CoV-2 infection follow the model described above, with an early EF response derived from differentiation of naïve B-cells to antibody-secreting plasmablasts, as well as pre-existing memory B-cells derived from seasonal HCoV exposure (247). Ongoing GC responses which produce long-lived SARS-CoV-2-specific plasma and memory cells are a feature of mild COVID-19. Studies demonstrate a progressive accumulation of V_H_ gene mutations in SARS-CoV-2 memory B cells (248), robust Tfh and GC responses in lymphoid tissues of infected patients at least 6 months post-infection (182), and the presence of long-lived plasma cells in the bone marrow of SARS-CoV-2 infected individuals (249). Underlying the ongoing GC response may be persistent SARS-CoV-2 antigenic exposure, as a recent study detected SARS-CoV-2 nucleic acids in the intestine of previously infected individuals for at least 3 months after infection (178).

Severe COVID-19 markedly alters the composition rather than quantity of the B-cell compartment of adaptive immunity. To this end, De Biasi et al. reported similar percentages of total B-cells and naïve B-cells in hospitalized COVID-19 patients and controls (250), which is consistent with other studies (251). However, while proportions of total and naïve B-cells among controls and patients were similar, transitional B-cell percentages were higher in COVID-19 patients (250, 251). Significantly, the absolute numbers and portions of memory switched and unswitched in COVID-19 patients were drastically decreased compared to healthy controls, and plasmablasts are expanded considerably in COVID-19 patients than in controls. Per these findings, other reports consistently show memory B-cell responses with amplification of antibody-secreting plasmablast responses in COVID-19 compared to healthy controls. A peculiar aspect of B-cell phenotypes in COVID-19 is the correlation of disease severity with values of peripheral DN B cells. Indeed, while patients and controls show comparable levels of total DN cells, DN subsets are profoundly altered in COVID-19. In particular, DN2 and DN3 are increased dramatically in severe and critical groups of COVID-19 (251, 252).

Furthermore, DN3 correlates with laboratory parameters of COVID-19 severity, such as arterial oxygen saturation. Although the function of DN B cells remains uncertain, studies reveal their expansion in various inflammatory and autoimmune diseases, such as systemic lupus erythematosus (SLE) (253). On the other hand, memory B cell responses inversely correlate with clinical severity and reduced hospitalization time (254).

Therefore, severe COVID-19 displays hallmarks of extrafollicular B-cell responses and higher neutralizing antibody titers, and high nAb titers correlate with inflammatory biomarkers, multi-organ failure, and mortality (252, 255). Consistently, individuals with lower IgG clear the virus better than stronger responders (256), suggesting that an exaggerated humoral response leads to the persistence of viral loads and disease exacerbation. However, why such an exaggerated nAb response in severe disease does not clear COVID-19 might be due to severely ill individuals failing to develop GC responses (257). Indeed, Tfh cells are markedly reduced in some patients’ draining LNs and spleen (258), which would also explain the low-level SHM characterizing the B-cell signature in advanced COVID-19. However, many individuals severely affected by COVID-19 develop potent Tfh responses, implying that they also generate GC responses but still suffer from severe COVID-19 (259). Dysregulated T-cell responses likely play a role in this, but further investigations are required to explain the apparent heterogenous immunologic phenotype observed in severe COVID-19 (260).

A macaque model of SARS-CoV infection posited antibody-dependent enhancement (ADE) as aggravating lung injury (261). However, these findings are complicated by data implicating a delayed, rather than amplified, IgG response in disease progression; indeed, IgA and IgM responses are roughly equivalent across disease severities (262, 263). Additionally, in severe patients, a redirection of the humoral immune response from the S-protein to the N-protein is observed, with S-predominant and N-predominant humoral responses in convalescent patients and deceased individuals, respectively (264). The potential role of ADE remains to be clearly defined, and contradictory data implicating a delayed and rewired humoral response in SARS-CoV-2 needs to be substantiated.

Intriguingly, the prevalence of autoantibodies – particularly against cytokines, chemokines, complement – is significantly higher in COVID-19 patients than in uninfected controls (265–267). Moreover, autoantibodies against tissue antigens correlate with disease severity (267), and the EF B cell repertoire in such settings resembles that seen in systemic lupus erythematosus (SLE) (164). Mainly, autoantibodies against type 1 IFNs are seen significantly more frequently in life-threatening COVID-19 patients than in patients with asymptomatic or mild COVID-19 and healthy controls, and these antibodies neutralize the interferon-mediated antiviral immune response *in vitro* (268). However, with the blunting of the IFN response caused by SARS-CoV-2 infection, the contribution of these anti-IFN autoantibodies remains undefined in severe COVID-19 cases.

Regarding the persistence of anti-SARS-CoV-2 humoral responses, a prospective study analyzing the longitudinal profile of serum anti-SARS-CoV-2 antibodies revealed a seroconversion rate of IgM, IgG, and IgA of 7-10 days PSO, with titers peaking at 4-6 weeks PSO (255, 269–271). Seroreversion – i.e., the time measured for serum anti-SARS-CoV-2 antibodies to wane – is rapid for IgM, with a median time of 7-10 weeks, but comparatively longer for IgG, which features modest declines after 5-8 months (178, 272). Current data is contradictory regarding the persistence of IgA (178). Therefore, although SARS-CoV-2 infection elicits robust B-cell responses described above, neutralizing antibodies wane rapidly and may not protect against reinfection (175, 273). These findings have rationalized postulations that booster vaccine doses may be necessary to sustain protective immunity, which is now being encouraged in parts of the world to combat mutant strains, such as the Omicron Variant. However, studies have revealed long-lasting SARS-CoV-2-specific memory B cells (175, 178, 274, 275). SARS-CoV-2-specific memory B cells are unchanged at six months PSO (178), and other studies show a continuous rise of anti-RBD and anti-N protein memory B cells at six (275) and eight months PSO (175), with a temporal switch from extrafollicular to germinal center maturation featuring somatic hypermutations in the variable region of anti-RBD Abs (247, 257). These findings hint at the potentially long-lasting efficacy of the current COVID-19 vaccines. Ongoing studies on the humoral trajectories of COVID-19 patients for extended periods will undoubtedly provide better insights into this topic

## 5 Conclusion

A remarkable undertaking from the scientific community has answered many pressing questions about the complex immunopathogenesis of COVID-19. We highlighted some of the current discussion points surrounding the immunopathogenesis of COVID-19. Future research on these issues will further our understanding of COVID-19 and potentially provide a foundation for developing interventions to combat this everchanging virus and help return to normalcy.

## Author Contributions

Conceptualization, AY, JK, and AS. Writing—original draft preparation, AZ, SS, and SA. Writing—review and editing, KK, AY, and JK. Supervision, AY and KK. Funding acquisition, KK. All authors have read and agreed to the published version of the manuscript

## Conflict of Interest

The authors declare that the research was conducted in the absence of any commercial or financial relationships that could be construed as a potential conflict of interest.

## Publisher’s Note

All claims expressed in this article are solely those of the authors and do not necessarily represent those of their affiliated organizations, or those of the publisher, the editors and the reviewers. Any product that may be evaluated in this article, or claim that may be made by its manufacturer, is not guaranteed or endorsed by the publisher.
